# Purification of Porcine Circovirus Type 2 Using an Affinity Chromatography Based on a Neutralizing Monoclonal Antibody against Viral Capsid Protein

**DOI:** 10.3390/pathogens10121564

**Published:** 2021-11-30

**Authors:** Haiqiao Bian, Chong Yu, Yanwu Wei, Li Feng, Changming Liu, Liping Huang

**Affiliations:** Swine Digestive System Infectious Diseases Research Team, State Key Laboratory of Veterinary Biotechnolgy, Harbin Veterinary Research Institute, Chinese Academy of Agricultural Sciences, Harbin 150069, China; bianhaiqiao@163.com (H.B.); yuchong06@163.com (C.Y.); weiyanwu@caas.cn (Y.W.); fengli@caas.cn (L.F.)

**Keywords:** porcine circovirus type 2, monoclonal antibody, affinity chromatography, virus purification

## Abstract

Porcine circovirus type 2 (PCV2) is a DNA virus without an envelope. The viral particle is icosahedral and has a diameter of approximately 17 nm. In order to obtain the purified virus, a broad-spectrum monoclonal antibody 3A5 against PCV2 was coupled to CNBr-activated Sepharose^TM^ 4B, and an affinity chromatography was established for PCV2 purification. A total of 6.5 mg of purified PCV2a/LG with 97% purity was obtained from 120 mL of the viral culture medium, and only PCV2 was detected by electron microscopy. No significant changes in the antigenic characteristics of the purified virus were detected by a capture enzyme-linked immunosorbent assay (ELISA). Furthermore, the titer of the purified PCV2 was 100 times higher than that of the unpurified virus. This affinity chromatography method was also used to purify PCV2b/LN590516 and PCV2d/SD446F16, and the purified viruses were detected by electron microscopy, capture ELISA, and virus titration, respectively. The results showed that these two strains can be successfully purified, but the yield is lower than that of the PCV2a strain. In addition, the purified virus could be used to study the viral adsorption and invasion of PK15 cells using indirect immunofluorescence assays. A large number of PCV2 signals were detected to transfer from the cellular surface to the periphery of the nucleus of the PK15 cells after 30 min of adsorption of the PCV2 to the PK15 cells. The affinity chromatography is a simple and convenient tool to obtain PCV2 with high purity. It could be applied for virus structure analysis, antibody preparation, and viral adsorption and invasion research.

## 1. Introduction

Porcine circovirus type 2 (PCV2) is a member of the genus *Circovirus* in the family *Circoviridae*. In 1991, the virus was isolated from pigs suffering from post-weaning multisystemic wasting syndrome in Canada [1]. Other diseases are also associated with PCV2 infection, including porcine reproductive failure, porcine respiratory disease complex, and granulomatous enteritis. These diseases have caused great economic losses to the pig industry worldwide. The diseases associated with PCV2 infection are defined by the American Porcine Veterinary Association as porcine circovirus-associated disease (PCVAD). In Europe, porcine circovirus disease (PCVD) is often used to refer to PCVAD [2].

The genome of PCV2 is circular, single-stranded, covalently closed, and composed of typical amphipathic DNA [3]. The genome is only 1766–1768 nt, containing five open reading frames (ORFs): ORF1, ORF2, ORF3, ORF4, and ORF5 [4,5,6,7,8,9,10]. Among them, ORF1 and ORF2 are relatively well studied. The former encodes virus replicase-related proteins (Rep and Rep’), and the latter encodes a virus capsid protein (Cap) [6,9,11]. ORF1 and ORF2 give PCV2 two essential features of a virus: the viral genome can be replicated and successfully assembled into mature progeny viruses. Based on the genetic evolution analysis of the entire PCV2 gene or the ORF2 sequence, PCV2 is currently divided into six genotypes, PCV2a–PCV2f [12,13], of which PCV2a, PCV2b, and PCV2d are the main genotypes [14,15]. Prior to 2003, PCV2a was the main epidemic genotype, whereas the PCV2b strain became the main epidemic strain beginning in 2004 [14]. Since 2006, the PCV2d strains have gradually spread and have become epidemic in many countries [14,16]. PCV2d–ORF2 shared 88.0–92.2% and 92.8–95.2% sequence identity with the ORF2 of PCV2a and PCV2b, respectively, while PCV2a–ORF2 and PCV2b–ORF2 shared 91.0–93.0% of the sequence identity [17].

PCV2 is a small unenveloped virus with 20-hedral symmetry (T = 1). Each virus particle consists of 60 CAP proteins, and the virus has a diameter of approximately 17 nm [3,18]. Cesium chloride (CsCl) gradient density centrifugation is commonly used to purify PCV2 [18,19,20]. There were also some chromatography methods for the virus purification. A three-step protocol was established for PCV2 VLPs purification from pupae, including extraction by detergent, ammonium sulfate precipitation, and anion exchange column chromatography [21]. Size exclusion chromatography was also used to purify the PCV2 recombinant CAP protein expressed in *Escherichia coli* [22]. Furthermore, a two-step chromatographic purification procedure of PCV2 CAP VLPs from yeast lysate was developed using Q Sepharose XL and cation-exchange CIMmultus SO3 monolith [23]. However, there has been no affinity chromatography reported for PCV2 purification until now.

In this study, we report an affinity chromatography based on a PCV2 broad-spectrum monoclonal antibody (mAb) to obtain PCV2 with high purity.

## 2. Results

### 2.1. Purification and Immobilization of PCV2 mAb 3A5

**Figure 1:**
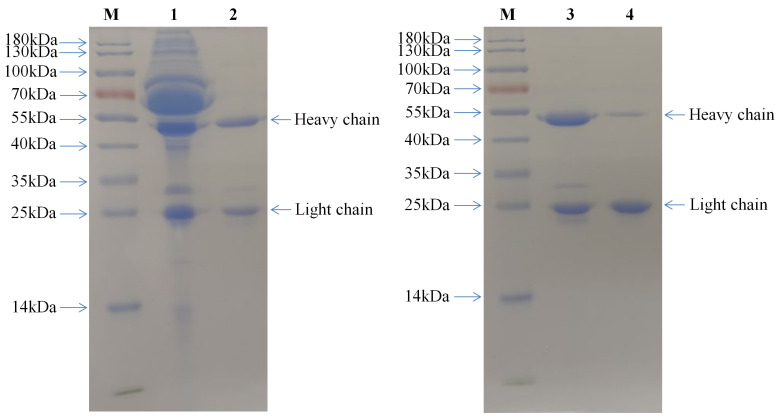
Coupling of PCV2 mAb 3A5 to CNBr-activated Sepharose^TM^ 4B. Unpurified and purified mAb 3A5 and its coupled Sepharose 4B were separated by SDS–PAGE. Lane M is a protein molecular weight marker. Lane 1 is unpurified mAb 3A5. Lane 2 and 3 are purified mAb 3A5. Lane 4 is mAb 3A5 coupled Sepharose 4B.

The mAb 3A5 was successfully purified using a protein G affinity chromatography column. A total of 26.5 mg of mAb 3A5 IgG was purified from 4 mL of ascites, with a purity of >94.6% (Figure 1). Twenty mg of purified IgG was coupled onto CNBr-activated Sepharose^TM^ 4B, and 20 μL coupled CNBr-activated Sepharose^TM^ 4B was used for the SDS–PAGE analysis. The results showed that the coupled Sepharose^TM^ 4B contained the light and heavy chains of the mAb 3A5 (Figure 1), indicating that mAb 3A5 IgG was successfully coupled onto Sepharose^TM^ 4B and could be used for subsequent PCV2 virus purification.

### 2.2. Purification of PCV2a/LG by the Affinity Chromatography Based on mAb 3A5

**Figure 2:**
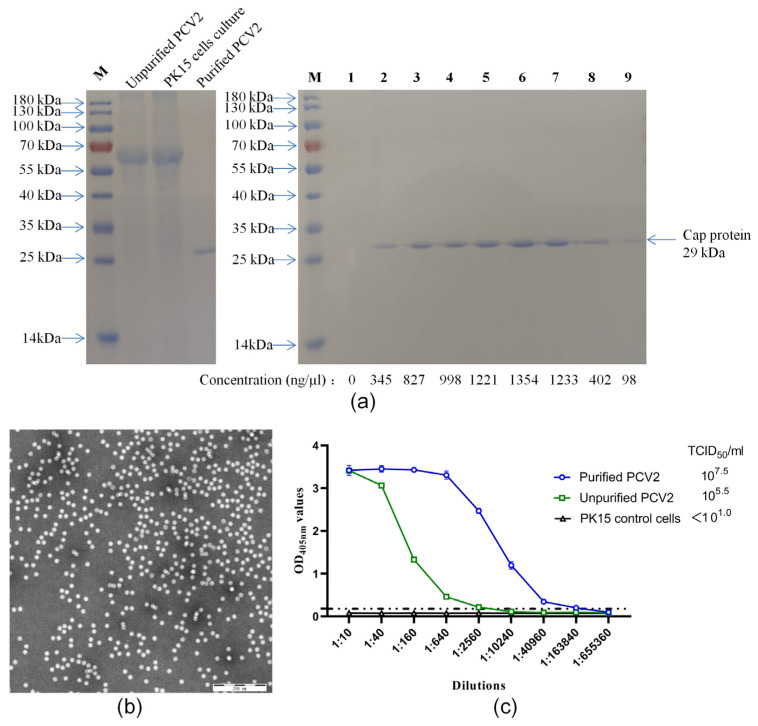
Identification of purified PCV2a/LG. (**a**) Unpurified and purified PCV2 were separated by SDS–PAGE. Lane M is a protein molecular weight marker. Lanes 1 to 9 are purified products from tubes no. 1 to 9. The concentrations of tubes no. 1–9 are marked below the lanes. (**b**) The purified virus was negatively stained with phosphotungstic acid and then observed by electron microscope. Bar = 40 nm. (**c**) Unpurified and purified PCV2 were tested by virus titration and the capture ELISA. Titers are shown on the top right corner. X-axis indicates the dilution ratios of the viruses and Y-axis shows the OD_405nm_ values. Dotted line indicates the cut-off value. Error bars indicate its SD.

A total of 120 mL of PCV2a/LG strain culture was purified using mAb 3A5-coupled Sepharose^TM^ 4B. The purified products were collected from 15 Eppendorf tubes, and their concentrations were measured using a NanoPhotometer^®^ NP80 (Implen GmbH, Munich, Germany). The concentrations of tube number one and tubes number 10 to number 15 were too low to be detected. The concentrations of tubes number two to nine were 345, 827, 998, 1221, 1354, 1233, 402, and 98 ng/µL respectively, as shown in Figure 2a. A total of 6.5 mg purified PCV2 was obtained. The purified PCV2 was assessed by an SDS–PAGE analysis, and only CAP protein (29 kDa) bands were found (Figure 2a). The purities of the target proteins were more than 97% using thin-layer scanning. These results indicated that the purified virus obtained by this method was highly pure. A sample with a concentration of 998 ng/µL was negatively stained with phosphomolybdic acid for electron microscopy. The results showed that the purified sample contained a large number of uniform virus particles with a diameter of approximately 17 nm, which was consistent with the morphology of PCV2 (Figure 2b). To test whether this purification method affected the infectivity of the virus, purified virus (998 ng/µL), unpurified PCV2 virus, and PK15 cell culture were tested for their virus titers. It was found that the virus titer of the purified virus reached 10^7.5^ median tissue culture infectious dose (TCID_50_)/mL, which was 100 times higher than that of the unpurified virus. No virus was detected in the PK15 cell culture samples. To test whether the reaction characteristics of the purified virus and mAb 3A5 had changed, capture ELISA was used to assess the ELISA titers of the purified and unpurified PCV2. The peak values (optical density at 405 nm, OD_405_) of the unpurified virus and purified virus were similar (Figure 2c). Because the titer of purified PCV2 was higher than that of the unpurified virus (Figure 2c), the capture ELISA titer of purified PCV2 was also higher than that of the unpurified virus (Figure 2c). We can infer that this affinity chromatography could be used to obtain highly pure PCV2, with no obvious influence on the morphology, antigenicity, or infectivity of the virus.

### 2.3. Purification of PCV2b and PCV2d Strains by the Affinity Chromatography Based on mAb 3A5

**Figure 3:**
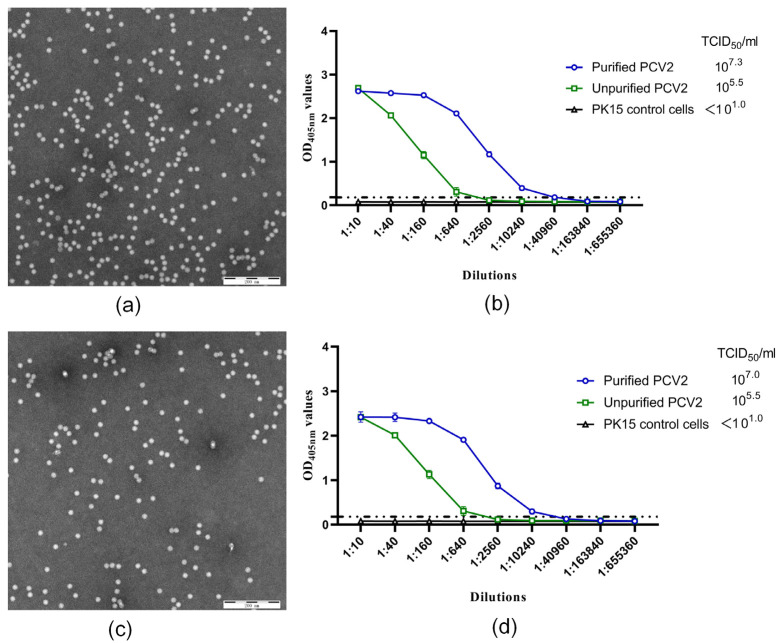
Identification of purified PCV2b/LN590516 and PCV2d/SD446F16. The purified PCV2b/LN590516 (**a**) and PCV2d/SD446F16 (**c**) were negatively stained with phosphotungstic acid and then observed by electron microscope. Bar = 40 nm. Unpurified and purified PCV2b/LN590516 (**b**) and PCV2d/SD446F16 (**d**) were tested by virus titration and the capture ELISA. Titers are shown on the top right corner. X-axis indicates the dilution ratios of the viruses and Y-axis shows the OD_405nm_ values. Dotted line indicates the cut-off value. Error bars indicate its SD.

To test whether this method could also be used for the purification of the PCV2b and PCV2d strains, we used the same method to purify 120 mL of PCV2b/LN590516 (10^6.0^TCID_50_/mL) and 120 mL of PCV2d/SD446F16 (10^6.0^TCID_50_/mL), respectively. The purified viruses were collected in ten tubes, respectively. The concentrations of the PCV2b/LN590516 products were 0, 212, 634, 763, 1034, 1067, 683, 302, 78, and 0 ng/µL, with a total amount of 4.8 mg. The concentrations of PCV2d/SD446F16 were 0, 137, 389, 624, 839, 1045, 936, 386, 57, and 0 ng/µL, with a total amount of 4.4 mg. The same virus purification products were mixed and detected for electron microscopy. The results showed that both the purified PCV2b/LN590516 and PCV2d/SD446F16 obtained pure virus particles, and there was no obvious contaminant protein (Figure 3a,c). At the same time, capture ELISA tests were performed on the purified samples, and the results showed that the ELISA titers of the purified PCV2b/LN590516 and PCV2d/SD446F16 were 40,960 and 10,240, respectively, which were higher than those of the unpurified viruses (Figure 3b,d). It could be concluded that this method can also be used for the purification of the PCV2b and PCV2d strains, but the recovery rate is 26.15%((6.5 − 4.8)/6.5 × 100%) and 32.31% ((6.5 − 4.4)/6.5 × 100%), lower than that of PCV2a, respectively.

### 2.4. PCV2 Adsorption and Invasion of PK15 Cells

**Figure 4:**
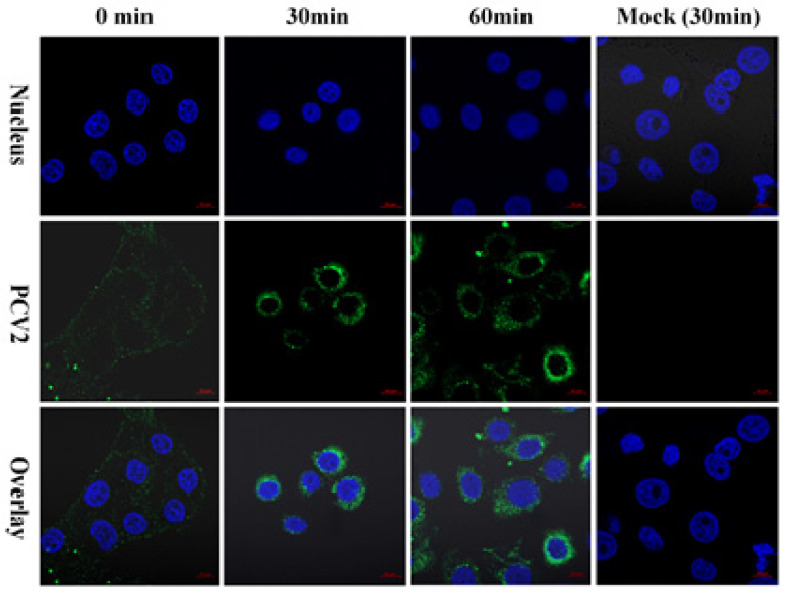
PCV2 adsorption and invasion of PK15 cells. PK15 cells were infected with purified PCV2a/LG at 4 °C for 1 h. After three washes with cold Minimum Essential Media (MEM; Invitrogen, Carlsbad, CA, USA), the cells were cultured in fresh MEM supplemented with 2% fetal bovine serum (FBS, Gibco, Grand Island, NY, USA) at 37 °C and were fixed at 0 min, 30 min, and 60 min with 100% cold methanol for 10 min at −20 °C. An IFA based on PCV2 mAb 8G12 was performed to stain PCV2 (green signals) as described previously [20]. Bar = 10 µm.

To test the adsorption of the PK15 cells by the PCV2, we adsorbed purified PCV2a/LG to PK15 cells at 4 °C, washed unadsorbed virus particles after 60 min, and traced the PCV2 by indirect immunofluorescence assay (IFA). A large number of PCV2 signals (green) were detected on the cell surface (Figure 4). To trace the PCV2 in the PK15 cells, IFA was also used to stain the PCV2 at 30 min and 60 min after the virus adsorption. Numerous PCV2 signals were detected around the nucleus from 30 min to 60 min (Figure 4). Therefore, when conducting research on the PCV2 invasion of susceptible cells, purified virus can be used to obtain clear and specific results.

## 3. Discussion

The virus purification technique is used in many applications, such as virus structure analysis, virus invasion analysis of host cells, virus-specific receptor identification, and specific antibody production [18,20]. As vaccine quality improves, the virus purification technique is becoming more widely used in vaccine production. Although CsCl gradient density centrifugation can be used to obtain higher concentrations and purity of virus particles, this method has some restrictions due to the lack of scalability and the enhancement of labor intensity in large-scale purifications. Chromatographic methods are regarded as an attractive alternative to ultracentrifugation [21,22,23]. The two-step chromatographic purification procedure of the PCV2 CAP VLPs from yeast lysate was developed using Q Sepharose XL and cation-exchange CIMmultus SO3 monolith, and PCV2 CAP VLPs with the purity of about 40% were obtained [23]. The PCV2 VLP expressed in *Escherichia coli* was purified by a single-column, high-throughput fractionation procedure based on size exclusion chromatography and the purity was 95% with a yield of 10 mg from 200 mL of bacterial culture [22]. A three-step protocol was established for PCV2 VLPs purification from pupae, including extraction by detergent, ammonium sulfate precipitation, and anion exchange column chromatography, and the final purity was about 90% with a yield of 4 mg from 10 pupae [21]. In this study, one-step affinity chromatography based on PCV2 neutralizing mAb and CNBr-activated Sepharose^TM^ 4B was established to purify PCV2. The yield was about 6.5 mg from 120 mL of PK15 cells culture with a purity of 97%. Compared with other non-specific methods of purifying PCV2, the affinity chromatography method established in this study was simpler in operation and the purified virus had high purity [21,22,23]. It is promising to be used in the production of PCV2 vaccines. In addition, the reproducibility of the affinity chromatography column needs to be further studied.

It was shown that the mAb 3A5 IgG was successfully coupled onto Sepharose^TM^ 4B, but the H chain of IgG was extremely reduced compared to the L chain (Figure 1). The possible reason was as following. The IgG was coupled to Sepharose^TM^ 4B by chemical bonds. The L or H chains coupled by chemical bonds could not be separated from the matrix by the sample processing method of the SDS–PAGE. As is well known, the L chain and H chain of IgG are connected by disulfide bonds. The L chain on the H chain or the H chain on the L chain can be detached from the matrix by SDS–PAGE. From the results of Figure 1, we could infer that the H chain of mAb 3A5 IgG is the main part coupled to the matrix.

Since PCV2 can tolerate an acidic environment with a pH of 3.0 [24], it is feasible that acidic elution is chosen in the purification process. As predicted, the purified virus still retains its original morphological characteristics and infectivity. Furthermore, a PCV2 particle is only 17 nm, and the large pore size of Sepharose^TM^ 4B provides sufficient space for PCV2 to bind to the mAb 3A5.

PCV2 VLPs were used in binding and internalization studies instead of PCV2 virions because of the difficulty in obtaining sufficient preparative amounts of the latter with high purity due to low PCV2 titers [25,26,27]. Fortunately, sufficiently purified PCV2 using the affinity chromatography based on mAb 3A5 was used to infect PK15 cells, and different internalization was observed as a result. The PCV2 VLPs were internalized slowly into the PK15 cells [25,27], while the PCV2 cells were internalized into the PK15 cells in 30 min in this study. From this result, we can infer that the internalization mechanism of the VLP without PCV2 nucleic acid cannot fully represent the true internalization mechanism of PCV2. Its internalization mechanism needs to be further studied.

In summary, this study used PCV2 monoclonal antibodies to establish a new affinity chromatography for PCV2 purification. This method does not require special equipment, is time-saving and simple to operate, and a virus can be obtained with high purity.

## 4. Materials and Methods

### 4.1. Cells, Viruses and Antibodies

PK15 cells (ATCC^®^ CCL-33^™^) free of PCV1 and PCV2 were grown in MEM (Invitrogen, Carlsbad, CA, USA) containing 10% heat-inactivated FBS (Gibco, Grand Island, NY, USA) for virus propagation and titration. PCV2a/LG strain, PCV2b/LN590516, and PCV2d/SD446F16 (MK347371) were used for purification, and their origins, genotypes, and accession numbers are shown in Table 1. Hybridomas secreting mAb 3A5, which neutralizes PCV2 strains from the PCV2a, PCV2b, and PCV2d genotypes, were cultured in Dulbecco’s Modified Eagle Medium (DMEM, Invitrogen) containing 10% FBS (Gibco) for mAb preparation [19,20]. 

### 4.2. Propagation of PCV2

The PCV2a/LG, PCV2b/LN590516, and PCV2d/SD446F16 (10^6^^.0^ TCID_50_/mL) were used as virus seeds to inoculate, respectively, freshly digested PK15 cell suspensions (2 × 10^5^ cells/mL) at a multiplicity of infection of 0.5. Viruses were maintained in MEM containing 2% FBS and 3 mM d-glucosamine (Sigma-Aldrich, St. Louis, MO, USA). After incubation at 37 °C for 120 h, the virus was collected from cultured infected cells by three freeze-thaw cycles and then centrifuged at 2500× *g* for 10 min at 4 °C. The supernatants containing the virus were aliquoted and stored at -80 °C. Virus titers were measured as described previously [28]. Samples with virus titers not less than 10^6.0^ TCID_50_/mL were used for virus purification.

### 4.3. Coupling of PCV2 mAb 3A5 to CNBr-Activated Sepharose^TM^ 4B

To obtain purified mAb 3A5, the ascites from BALB/c mice containing mAb 3A5 were produced as described previously [29] and were purified using protein G Sepharose^TM^ CL-4B (GE Healthcare, Uppsala, Sweden) according to the manufacturer’s instructions. The concentration of purified mAb was measured using a NanoPhotometer^®^ NP80 (Implen GmbH, Munich, Germany), and its purity was analyzed by SDS–PAGE, followed by thin-layer scanning [29]. A total of 20 mg purified IgG was dialyzed using coupling buffer (0.1 M NaHCO_3_ solution containing 0.5 M NaCl, pH 8.3) at 4 °C for 18 h and then coupled to CNBr-activated Sepharose^TM^ 4B (GE Healthcare) according to the manufacturer’s instructions.

### 4.4. Purification and Evaluation of PCV2a Strain

A total of 120 mL PCV2a/LG (10^6.0^ TCID_50_/mL) was centrifuged at 6800× *g* for 30 min, and the supernatant was filtered using a 0.45 μm filter. One milliliter of the coupled matrix was added to the clarified virus solution, and the mixture was rotated end-over-end overnight at 4 °C. The mixture was loaded onto the column (Sigma-Aldrich, St. Louis, MO, USA) under gravity several times. To remove any proteins that were not bound to mAb 3A5, the column was fixed vertically and washed with 20 column volumes of washing buffer (20 mM NaHPO_4_, 0.5 M NaCl, pH 7.4). PCV2a/LG was eluted from the column using elution buffer (0.1 M glycine-HCl, pH 3.0) into Eppendorf tubes containing 100 µL of 1 M Tris-HCl (pH 8.0) per 1 mL eluent. The concentrations of purified PCV2 were measured using a NanoPhotometer^®^ NP80 (Implen GmbH, Munich, Germany).

To test its purity, the purified PCV2a/LG was mixed with an equal volume of Laemmli buffer (2×), boiled for 5 min, and separated by standard SDS–PAGE, followed by thin-layer scanning [29]. The purified virus (998 ng/µL) was negatively stained with 3% phosphotungstic acid (pH 7.2), and the virus suspension was placed on a 400 mesh grid. The virus particles were observed using a Hitachi H-7650 electron microscope (Hitachi, Ltd. Tokyo, Japan).

Capture ELISA based on mAb 3A5 was used to test the reactivity of the purified PCV2 with its antibody as described previously [19]. Purified virus (998 ng/µL) and unpurified virus were serially diluted four-fold from 1:10 to 1:163840 with 1% bovine serum albumin-phosphate buffered saline (PBS) and were measured using the capture ELISA [19]. Experiments were repeated in triplicate. At OD_405_ values ≥ 0.18, the sample was considered to be positive. At 0.15 < OD_405_ < 0.18, tests were repeated. At OD_405_ < 0.15, the sample was considered negative.

To test whether the purification process affected the infectivity of purified PCV2, purified PCV2 (998 ng/µL) and unpurified virus were detected using virus titration as described previously [28].

### 4.5. Purification and Evaluation of PCV2b and PCV2d Strains

Since the monoclonal antibody 3A5 can not only react with the PCV2a strain but also bind to the PCV2b and PCV2d strains, can the affinity chromatography method based on this mAb also be used to purify the PCV2b and PCV2d strains? Based on the above problems, 120 mL PCV2b/LN590516 strain (10^6.0^TCID_50_/mL) and 120 mL PCV2d/SD446F16 strain (10^6.0^TCID_50_/mL) were purified using the same chromatography column and method, and then UV method, capture ELISA, virus titration, and electron microscope were used to detect the concentration, reactivity, virus titer, and purity of the purified virus.

### 4.6. Assessment of Adsorption and Invasion of PCV2 Using Purified Virus

To assess the adsorption and invasion of PCV2 to PK15 cells, the cells at 80% confluency were infected with purified PCV2a/LG (200 ng/µL) and incubated at 4 °C for 1 h. After three washes with cold MEM, the cells were cultured in fresh MEM supplemented with 2% FBS at 37 °C and were fixed at 0 min, 30 min, and 60 min with 100% cold methanol for 10 min at −20 °C. An IFA was performed to stain PCV2 as described previously [14]. The mAb 8G12 against PCV2 CAP [19] and fluorescein isothiocyanate-conjugated goat anti-mouse IgG1 (1:400 in PBS; Life technologies, Eugene, OR, USA) were used as the primary and secondary antibodies, respectively. To visualize nuclei, the cells were stained with 4’,6-diamidino-2-phenylindole, and stained cells were analyzed by fluorescence confocal microscopy (LSM 800 confocal laser scanning microscope with Airyscan; Zeiss, Oberkochen, Germany).

## Figures and Tables

**Table 1:** Origins of the PCV2 strains used in this study.

Isolates Name	Isolate Region	Isolate Time	Genotype	Genome Length (nt)	Accession Numbers
LG	Jilin	2008	PCV2a	1768	HM038034
LN590516	Liaoning	2016	PCV2b	1767	MK347352
SD446F16	Shandong	2019	PCV2d	1767	MK347371

## Data Availability

Data sharing is not applicable to this article.
